# Impact of Notch disruption on myeloid development

**DOI:** 10.1038/bcj.2017.73

**Published:** 2017-08-25

**Authors:** O L Francis, K K Chaudhry, T Lamprecht, J M Klco

**Affiliations:** 1 Department of Pathology, St. Jude Children’s Research Hospital, Memphis, TN, USA

The Notch pathway is a conserved signaling network that regulates many cellular processes including renewal of stem cells, differentiation of multiple cell lineages, proliferation and apoptosis.^1^ Notch signaling involves the binding of Notch ligands to Notch receptors followed by proteolytic cleavage events, translocation of intracellular Notch (ICN) to the nucleus and regulation of target genes via the interaction of transcription factor *CSL/RBPJ* and the MAML family of transcriptional co-activators.^1, 2^ The Notch pathway is involved in lymphoid development and recurrent activating mutations in *NOTCH1* contribute to T lymphoblastic leukemias.^3^ Whether Notch signaling is actively involved in the regulation of myeloid development and myeloid leukemogenesis is less clear due to conflicting reports.^4, 5, 6^ In this study, we abrogated canonical Notch signaling throughout the hematopoietic system to evaluate the role of Notch in myelopoiesis and to conclusively determine if inhibition of Notch signaling can contribute to aberrant myelopoiesis and lead to the development of a myeloid neoplasm.

To abrogate Notch signaling, we utilized the well-studied conditional DN-MAML1-GFP mouse model previously shown to block canonical Notch signaling via inhibition of Notch receptors 1–4.^7^ We disrupted Notch signaling throughout the hematopoietic system, including myeloid stem and progenitor cells, by intercrossing DN-MAML1-GFP mice with Vav-cre mice.^8^ The DN-MAML1-GFP is a fusion protein; thus evaluation of GFP levels by flow cytometry was used to track cells expressing DN-MAML1. As expected, doubly heterozygous mice demonstrated GFP expression in most of the cells in the bone marrow and to a lesser extent in the spleen and thymus (Figure 1a; Supplementary Figure 1). Some mice demonstrated a notable reduction of DN-MAML1 expressing cells only in the thymus, suggesting there is strong selective pressure in T cells to not express DN-MAML1.

To validate our mouse model, we first determined if canonical Notch signaling is decreased in mice doubly heterozygous for DN-MAML1-GFP and Vav-cre (DNM^f/−^Vav^+/−^). The development of marginal zone (MZ) B-cells in the spleen relies on Notch 2 signaling and Notch blockade results in the reduction of the MZ B-cell pool in murine spleens.^7^ As shown in Figures 1b and c, a significant reduction in the percentage of MZ B-cells was observed in DNM^f/−^Vav^+/−^ mice compared to controls at 6 months. Second, we confirmed that these mice exhibited the expected abnormalities in thymocyte development, a Notch1 process.^9^ We found a significant increase in the double negative (DN) population within the GFP+ fraction of thymocytes taken from DNM^f/−^Vav^+/−^ mice compared to controls (Figure 1d). Further evaluation of the DN thymocyte populations in DNM^f/−^Vav^+/−^ mice showed a significant increase in the frequency of the more immature DN1/2 cells, a decrease in DN3/4 cells (Figure 1e) and an increase in B220+ B cells (Figure 1f). Taken together, these data provide confirmatory evidence that our *in vivo* model is sufficient to inhibit canonical Notch signaling over time.

Earlier studies suggest that loss of Notch signaling can impair megakaryopoiesis leading to a decrease in megakaryocyte–erythroid progenitors (MEPs) at the expense of an increase in granulocyte–monocyte progenitors (GMPs).^10^ To determine whether loss of Notch signaling in our model affects the myeloid progenitor pool, we analyzed stem and progenitor compartments of control and DNM^f/−^Vav^+/−^ mice at 6 months and 15–18 months. Analyses of DNM^f/−^Vav^+/−^ mice at 15–18 months revealed a trend toward an increase in myeloid progenitors (Figure 2a), a significant increase in GMPs (Figure 2b) and a significant decrease in CMPs (Figure 2b). There was, however, no significant difference in the LSK (Figure 2a) or the CD150+CD48− LT-HSC populations (data not shown). A similar trend was observed in the spleens of these mice along with an increase in CD11b+ cells (Supplementary Figures 2A–E). This significant increase in GMPs was observed as early as 6 months (Supplementary Figure 3). Finally, bone marrow cells from mice expressing DN-MAML1-GFP produced fewer myeloid colonies in methylcellulose at 6 months (Supplementary Figure 4) compared to age-matched controls. The collective data suggest that loss of Notch signaling contributes to a decrease in CMPs and a mild but stable expansion of the GMP and myeloid compartments.

Previous results from the disruption of Nicastrin showing marked expansion of myeloid cells prompted speculation that Notch functions as a tumor suppressor.^5^ To determine if canonical Notch signaling can function as a tumor suppressor in myelopoiesis, a tumor watch was established (*n*=19 DNM^f/−^Vav^+/^^−^; *n*=15 controls). The mice were aged to 15–18 months and there was no evidence of a highly penetrant myeloid disease with loss of canonical Notch signaling as shown by no significant difference in survival (Figure 2c), immunophenotype of the peripheral blood (Figure 2d) and white blood cell count between the two groups (Figure 2e). At the end point, the collective DN-MAML1 cohort also did not have splenomegaly (Figure 2f). During the course of the experiment, two mice died in the DNM^f/−^Vav^+/−^ group near the end of the study; one had an enlarged spleen (0.510 g) but viable cells could not be obtained and the other died abruptly without splenomegaly. A single mouse in the control group also died abruptly without splenomegaly. These mice continued to show >90% GFP expression at 12 months and decreased MZ B-cells at 18 months (Supplementary Figures 5A–C), demonstrating that there was continued abrogation of Notch signaling. The above data suggest that although there is an expansion of GMPs and myeloid cells in mice that lack canonical Notch signaling, these changes are not sufficient to produce a myeloid neoplasm. It is possible that loss of Notch signaling in myeloid cells is insufficient for the development of myeloid neoplasms, but may cooperate with other events such as genes frequently mutated in AML.^11^ To test this hypothesis, we retrovirally expressed FLT3^ITD^ (MSCV-Ires-mCherry) in control and DNM^f/−^Vav^+/−^ lineage negative bone marrow cells followed by transplantation into syngeneic recipients (Figure 2g; Supplementary Figure 6A). By ~150 days, all control-FLT3^ITD^ mice died, while there was an observed delay in disease progression and subsequent death of DNM^f/−^Vav^+/−^ FLT3^ITD^ mice (Figure 2h). There was a significant reduction in the spleen weights (Figure 2i) and frequency of mCherry+ cells in the spleen of DNM^f/−^Vav^+/−^ FLT3^ITD^ mice (Figure 2j). Immunophenotyping of splenocytes showed no significant differences within the myeloid lineage (Figure 2k). Further, *in vitro* methylcellulose experiments performed using Lin-marrow cells from both groups transduced with FLT3^ITD^ mCherry construct showed no advantage in growth of DNM^f/−^Vav^+/−^ FLT3^ITD^ compared to control-FLT3^ITD^cells (Supplementary Figure 6B). Overall, these data suggest that loss of Notch signaling does not cooperate *in vivo* with FLT3^ITD^ to induce a myeloid disease.

The results from our study demonstrate that prolonged loss of Notch signaling *in vivo* does not lead to a highly penetrant myeloid neoplasm. We found that expression of DN-MAML1-GFP in bone marrow cells, including hematopoietic stem and progenitor cells (HSPCs), resulted in a relative increase in GMPs that was stable over time and an increase in myeloid cells in the spleen. Despite these observations, there were no overt clinical features consistent with the development of a myeloid neoplasm. Our results are consistent with other studies that evaluated the impact of Notch inhibition on normal hematopoiesis via expression of DN-MAML1 or conditional ablation of the *Rbpj* gene.^4^ Additionally, our results regarding the impact of Notch blockade on leukemia growth using the FLT3^ITD^ model is congruent with our previous data, which demonstrated that inhibition of Notch signaling, via retroviral expression of DN-MAML1 or treatment with gamma secretase inhibitors, can inhibit leukemia growth.^6^ Furthermore, inactivating mutations in the Notch pathway are uncommon in primary myeloid neoplasms.^12^ These studies are in contrast to the results from disruption of Nicastrin, which led to a marked expansion of myeloid cells and a fully penetrant myeloid neoplasm at 5 months, prompting speculation that Notch functions as a tumor suppressor in myeloid cells.^5^

These differences are likely multifactorial and include cell context, the varying contribution of intrinsic and extrinsic signaling and dosage of pathway modulation; altogether highlighting the complexity of the Notch pathway. To achieve loss of Notch1-4 signaling *in vivo,* we utilized the dominant-negative mastermind-like transgene in which the ICN transcriptional co-activator MAML1 is truncated and fused to GFP^7^ preventing the formation of the ternary complex that is required for the transcriptional activation of Notch target genes. This approach should yield a more specific disruption of canonical Notch signaling than deleting Nicastrin, which has other substrates besides Notch. Finally, we avoided the Mx-1 cre system, and relied on the more hematopoietic-specific Vav-cre model. Not only is the Mx-1-Cre transgene active in mesenchymal stem cells in the bone marrow stroma,^13^ but it requires an interferon response for induction and it’s well known that interferon signaling can alter stem cell function and induce proliferation of myeloid cells.^14^ Collectively, these features may lead to cell extrinsic effects on myeloid development and leukemogenesis.

Altogether, our results demonstrate that loss of Notch signaling *in vivo* is insufficient for the development of myeloid neoplasms. This lack of a strong phenotype observed after Notch blockade on normal hematopoiesis is encouraging for clinical studies evaluating the impact of different Notch pathway inhibitors on both solid and liquid tumors.^15^ Furthermore, our results highlight the importance of using selective *in vivo* mouse models of Notch perturbation for future evaluation of the underlying mechanisms of Notch signaling in myeloid malignancies.

## Figures and Tables

**Figure 1:**
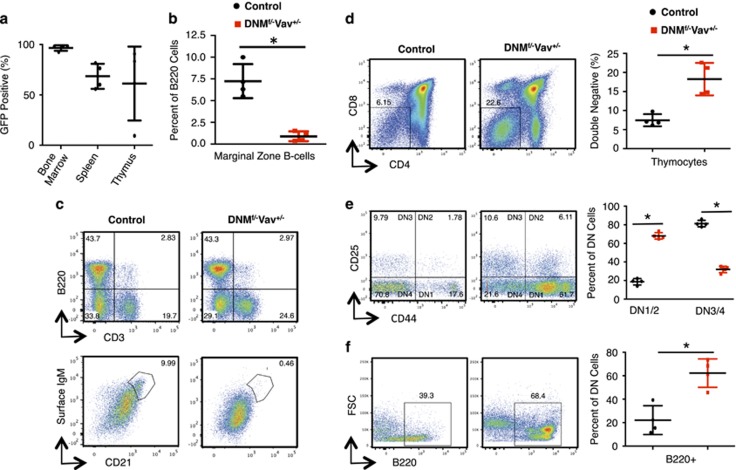
DN-MAML1-GFP × Vav-cre (DNM^f/−^Vav^+/−^) mice have reduced marginal zone B-cells and increased immature T-cells. (**a**) DNMAML1 (GFP+) cells found in the bone marrow, spleen and thymus of DNM^f/−^Vav^+/−^ mice were quantified using flow cytometry at 6 months (*n*=4). (**b**) MZ B-cells harvested from the spleens of control and DNM^f/−^Vav^+/−^ mice were quantified using flow cytometry at 6 months (*n*=4). (**c**) Representative dot plots of MZ B-cells identified by gating on B220+CD3− cells (top panels), followed by sIgM+CD21+ cells (bottom panels). (**d**) Representative dot plots of double negative (DN) thymocytes isolated from DNM^f/−^Vav^+/−^ and control mice at 6 months are identified as CD4−CD8− cells using flow cytometry. (**e**) Representative dot plots of DN subsets identified as CD4−CD8−CD25−CD44+ (DN1), CD4−CD8−CD25+CD44+ (DN2), CD4−CD8−CD25+CD44− (DN3) and CD4−CD8−CD25−CD44− (DN4) populations. (**f**) Additional analyses of DN cells using B-cell marker B220. Quantification of data from four mice for Figures 1d–f is shown in the right panels. Data are representative of two or more independent experiments. Statistical significance was determined by a two-tailed, unpaired *t-*test (*P⩽0.05).

**Figure 2:**
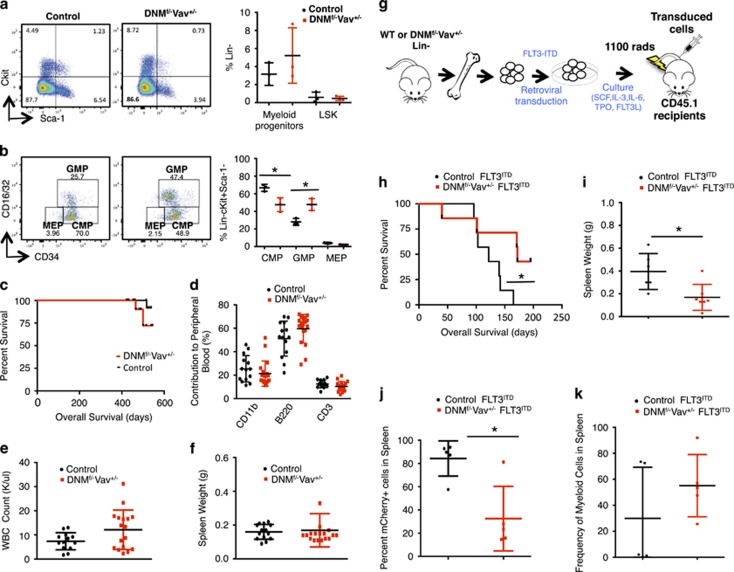
Long-term inhibition of Notch signaling expands the GMP compartment but does not produce highly penetrant myeloid neoplasms. (**a**) HSPC analysis of bone marrow cells harvested from DNM^f/−^Vav^+/−^ and control mice killed at 15–18 months. Representative dot plots showing DNM^f/−^Vav^+/−^ LSK cells and total myeloid progenitors. DNM^f/−^Vav^+/−^ HSPCs were identified by first gating on the GFP+ fraction of Lin-negative cells, followed by Sca-1+cKit+ cells (LSK) or Sca-1-cKit+ cells (total myeloid progenitors). (**b**) DNM^f/−^Vav^+/−^ myeloid progenitor subsets were identified by gating on the GFP+ fraction of Lin-negative cells, followed by cKit+Sca-1- and CD16/32 versus CD34: CMP (Lin-Sca-1-cKit+CD16/32−CD34+), GMP (Lin-Sca-1-cKit+CD16/32+CD34+) and megakaryocyte–erythroid progenitors (MEP) (Lin-Sca-1-cKit+CD16/32−CD34−). Representative dot plots of HSPC analyses are shown in Figures 2a and b (left panels) and the quantification of data from three mice are graphed (right panels). (**c**) Kaplan–Meier plot of the survival of DNM^f/−^Vav^+/−^ (*n*=19) and control (*n*=15) mice aged to 15–18 months. (**d**) Flow cytometric analysis of lineage subsets (Myeloid, B- and T-cells) in peripheral blood taken from DNM^f/−^Vav^+/−^ (*n*=19) and control (*n*=14) mice at 18 months. (**e**) Complete blood counts were also performed using peripheral blood collected from mice at 18 months in order to compare the total number of WBCs in DNM^f/−^Vav^+/−^ (*n*=17) and control (*n*=13) mice. (**f**) At 15–18 months, mice were killed, spleens were harvested and spleen weights were compared in the graph: DNM^f/−^Vav^+/−^ (*n*=18); controls (*n*=14). (**g**) Donor BM cells were harvested from DNM^f/−^Vav^+/−^ or control mice at killing, Lin- cells were transduced with FLT3^ITD^ MSCV-Ires-mCherry (MIC) construct and transplanted into CD45.1 recipients. (**h**) Kaplan–Meier plot of the survival of DNM^f/−^Vav^+/−^ FLT3^ITD^ (*n*=7) and control-FLT3^ITD^ (*n*=7) mice. (**i**) Spleen weights of DNM^f/−^Vav^+/−^FLT3^ITD^(*n*=7) and control-FLT3^ITD^ (*n*=6) mice. (**j**) Frequency of FLT3^ITD^ cells in the spleens of mice was determined by assessing mCherry levels using flow cytometry. (**k**) Immunophenotype of splenocytes from DNM^f/−^Vav^+/−^FLT3^ITD^ (*n*=5) mice compared to control-FLT3^ITD^ (*n*=5) was determined by staining samples with myeloid lineage markers (CD11b, Gr-1) and analyzing samples with flow cytometry. Statistical significance was determined by a two-tailed, unpaired *t-*test (*P⩽0.05).
