# Supplementary material for: Impact of Notch disruption on myeloid development
Source: Blood Cancer J. 2017 Aug 25;7(8):e598–. doi: 10.1038/bcj.2017.73 (PMC5596384; doi:10.1038/bcj.2017.73)
Supplement: Supplementary Information [file bcj201773x1.docx]

**Supplementary Information**

**Impact of Notch Disruption on Myeloid Development**

Olivia L. Francis^1^, Kamaljit Chaudhry^1^, Tamara Lamprecht^1^, Jeffery M. Klco^1*^

This file contains Supplementary Materials and Methods, Supplementary References and Supplementary Figures with legends.

**Supplementary Materials and Methods**

***Mice***

All animal procedures were approved by the Institutional Animal Care and Use Committee of St. Jude Children’s Research Hospital or by the Washington University Animal Studies Committee. DNMAML1-GFP ^f/-^ (ROSA ^DNMAML+/-^) mice were generated as described by Tu *et. al*^(1)^ and were a kind gift of Dr. Warren Pear (University of Pennsylvania, School of Medicine, PA, USA). Vav-iCre^+/-^ mice described in Georgiades,^(2)^ et al were obtained from the Jackson Laboratory, Bar Harbor, ME, USA. Doubly heterozygous mice (DNM^f/-^Vav^+/-^) that lack Notch signaling in the hematopoietic compartment, were generated by crossing DNMAML1-GFP ^f/-^ mice with Vav-iCre^+/-^ mice. For all studies control mice are single heterozygous offspring from the above mating strategy.

***Genotyping***

The genotype of DNMAML1 x Vav-cre progeny were confirmed by digesting the toes of <10 day old pups and performing KlenTaq LA PCR amplification using the Rosa 1 (AAAGTCGCTCTGAGTTGTTAT), Rosa 2 (GCGAAGAGTTTGTCCTCAACC) and Rosa 3 (GGAGCGGGAGAAATGGATATG) primer sequences to confirm DNMAML1; and the Vav transgene’s forward (AGATGCCAGGACATCAGGAACCTG) and reverse (ATCAGCCACACCAGACACAGAGATC) sequences to confirm Vav-cre. Gel electrophoresis was used to visualize the PCR amplicon.

***Hematopoietic tissue processing***

On day of euthanasia, single cell suspension was obtained from the long bones, spleen, thymus and peripheral blood of mice. Bone marrow cells were obtained by flushing the long bones with PBS using a 27½ g syringe (BD Biosciences) and white blood cells were obtained after lysing red blood cells with ACK lysis buffer (Gibco). Single cell suspensions were obtained from spleen and thymus by crushing tissues with a syringe plunger and filtering through a 70 micron cell strainer (Fischer Scientific).

***DNA constructs and retrovirus production***

The pMSCV-IRES-FLT3^ITD^-GFP construct was a kind gift from Charles Mullighan (St. Jude Children’s Research Hospital, Memphis, TN, USA). pMSCV-IRES-FLT3^ITD^-mCherry was generated by subcloning FLT3^ITD^ into pMSCV-IRES-mCherry vector. Retrovirus was produced by co-transfecting 293T cells with pMSCV-IRES-FLT3^ITD^-mCherry and an ecotropic packaging vector (Ecopac) as previously described^(3)^, using Fugene HD transfection reagent (Promega, Madison, WI, USA).

***Retroviral transduction and bone marrow transplantation***

Lineage negative (Lin-) cells isolated from whole bone marrow cells that were collected from the femurs and tibias of DNM^f/-^Vav^+/-^ or control mice were cultured in media containing RPMI, Pen/Strep, interleukin-3 (10ng/ml), interleukin-6 (10ng/ml), thrombopoietin (10ng/ml), stem cell factor (100ng/ml) and Flt3 ligand (50ng/ml) (Peprotech, Rocky Hill, NJ, USA) overnight followed by transduction (x 2) using retroviral supernatant from pMSCV-IRES-FLT3^ITD^-mCherry infected 293T cells. Syngeneic recipient mice irradiated with a lethal dose of 1100 rads were transplanted intravenously with 0.5x10^6^ DNM^f/-^Vav^+/-^ or 0.5x10^6^ control Lin- donor cells that were transduced with FLT3^ITD^ (~10% transduction efficiency).

***Flow cytometry***

DNMAML1 expression was evaluated by assessing GFP positivity (DN-MAML1-GFP is a fusion product) in cells of hematopoietic tissues (bone marrow, spleen, thymus and peripheral blood) using flow cytometry. Cells were harvested from bone marrow, spleen and peripheral blood and stained with specific anti-mouse antibody clones listed in Supplementary Table 1 to evaluate the frequency of hematopoietic stem and progenitors (HSPCs), marginal zone (MZ) B-cells, T-cells and myeloid cells. Cells were incubated with antibodies on ice for 20 minutes and the fixable viability stain 620 (BD BioSciences, San Jose, CA, USA) was used to assess cell viability. Fluorescently labeled cells were analyzed using the LSRFortessa (BD Biosciences, San Jose, CA, USA) and Flowjo analysis software (Flowjo LLC, Ashland, OR, USA). Gates were set based on control samples including unstained, isotype control or fluorescence minus one (FMOs).

***Immunophenotyping of leukemic samples***

Tissues were harvested and processed before (peripheral blood) or after death (spleen and bone marrow) and cells were stained with a panel of stem cell and lineage markers (Supplementary Table 1). Samples were analyzed using the LSRFortessa (BD Biosciences) and flowjo (Flowjo, LLC).

***Complete blood counts***

Peripheral blood complete blood counts were performed using the FORCYTE^TM^ (Oxford Science Inc.) by the St. Jude Children’s Research Hospital’s Veterinary Pathology Core.

***Methylcellulose colony-forming assay***

Myeloid colony generation was assessed by plating 10,000 DNMAML1-GFP or control whole bone marrow cells (in triplicate) in 1ml of methylcellulose-based complete medium (M3434, Stem Cell Technologies, Vancouver, BC, Canada). Cells were cultured in a 35-mm culture dish and incubated in a 5% CO_2_ incubator at 37^o^C for 7 days. Self-renewal was evaluated by re-plating cells harvested on day 7 in methylcellulose-based complete medium for another 7 days. This procedure was repeated for several weeks (serial re-plating). Similar experiments were performed using DNM^f/-^Vav^+/-^ FLT3^ITD^ and control-FLT3^ITD^ cells.

***Statistical analyses***

Statistical analyses were performed using Graphpad Prism and are shown as means ± SD (standard deviation). Animal survival data was generated using Kaplan-Meier plots in GraphPad Prism (GraphPad Software Inc, La Jolla, CA, USA). Experimental group comparisons were evaluated using the two-tailed, unpaired *t*-test in GraphPad InStat (GraphPad Software Inc, La Jolla, CA USA).

**Supplementary Table 1:**

| **Cell Populations** | **Schema** | **Antibodies** | **Clone #** | **Company** |
| --- | --- | --- | --- | --- |
| HSPCs | Lin | Ter119 Biotin | Ter-119 | EBioscience |
|  |  | CD11b Biotin | M1/70 | EBioscience |
|  |  | CD3 Biotin | 145-2C11 | EBioscience |
|  |  | CD4 Biotin | GK1.5 | EBioscience |
|  |  | Gr-1 Biotin | RB6-8C5 | EBioscience |
|  |  | B220 Biotin | RA3-6B2 | EBioscience |
|  |  | Streptavidin-BV711 | N/A | BioLegend |
|  | LSK | Lin cocktail | N/A | - |
|  |  | cKit APCefluor780 | 2B8 | EBioscience |
|  |  | Sca-1 PerCP-Cy5.5 | D7 | EBioscience |
|  | LSK-SLAM | LSK cocktail | N/A | - |
|  |  | CD150 BV605 | TC15-12F12.2 | BioLegend |
|  |  | CD48 PECy7 | HM48-1 | EBioscience |
|  | Myeloid Progenitors | LSK | N/A | - |
|  |  | FcγR II/III efluor450 | 93 | EBioscience |
|  |  | CD34 efluor660 | RAM34 | EBioscience |
| B-cells | B-cells | B220 APCefluor780 | RA3-6B2 | EBioscience |
|  | MZ B-cells | sIgM APC | II/41 | EBioscience |
|  |  | CD21/CD35 efluor450 | eBio4E3 | EBioscience |
| T-cells | T-cells | CD3 PerCPCy5.5 | 145-2C11 | EBioscience |
|  | Mature  T- cells | CD4 efluor450 | GK1.5 | EBioscience |
|  |  | CD8 APC | 53-6.7 | EBioscience |
|  | Immature  T-cells  (DN1-DN4) | CD25 PE | PC61.5 | EBioscience |
|  |  | CD44 PECy7 | IM7 | EBioscience |
| Myeloid cells | Granulocyte/  Monocyte | CD11b efluor450 | M1/70 | EBioscience |
|  |  | Gr-1 PECy7 | RB6-8C5 | EBioscience |
| Leukemia  Immunophenotype | Immature cells | cKit APCefluor780 | 2B8 | EBioscience |
|  |  | CD34 efluor660 | RAM34 | EBioscience |
|  | Mature cells | B220 BV711 | RA3-6B2 | BioLegend |
|  |  | CD3 PerCPCy5.5 | 145-2C11 | EBioscience |
|  |  | CD11b eVolve | M1/70 | EBioscience |
|  |  | Gr-1 PECy7 | RB6-8C5 | EBioscience |
|  | White Blood Cells | CD45.2 efluor450 | 104 | EBioscience |

**Supplementary Figures:**


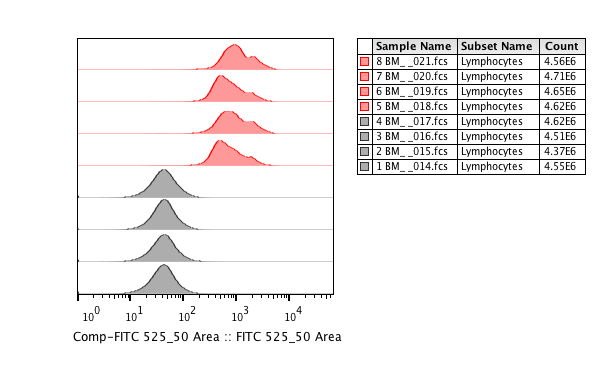

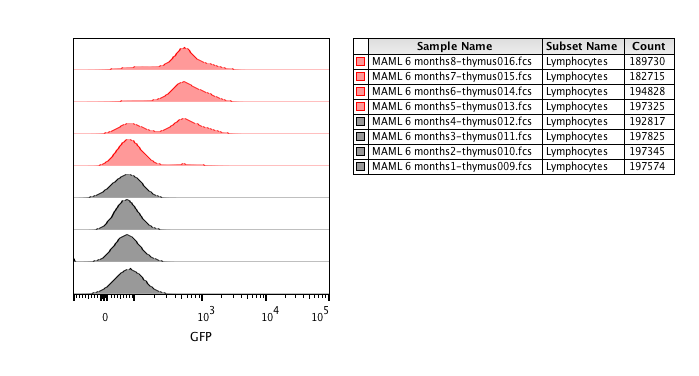

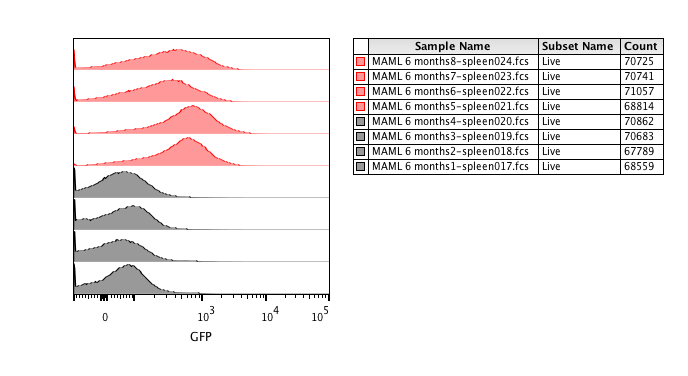


**Bone Marrow**

**GFP**

**Spleen**

**Thymus**

DNM^f/-^Vav^+/-^

Control

**Count**

**Supplementary Figure 1: Frequency of DNMAML1 cells in the hematopoietic tissues of DNM^f/-^Vav^+/-^ mice at 6 months**. DNMAML1 cells (red) found in the bone marrow, spleen and thymus of DNM^f/-^Vav^+/-^ mice were evaluated for GFP expression at 6 months using flow cytometry (n=4). Cells from control mice are also shown (grey)(n=4).


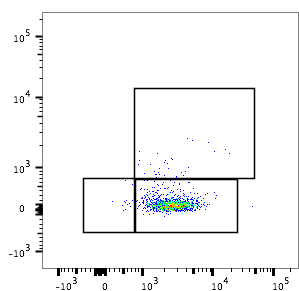

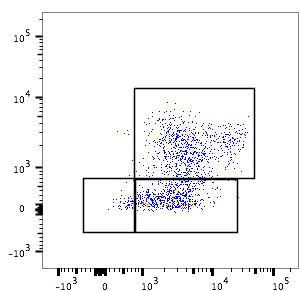

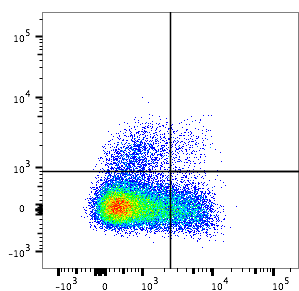

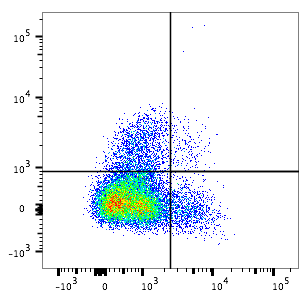


% Lin-

Myeloid

progenitors

LSK

**Control**

**DNM^f/-^Vav^+/-^**

1.18

6.64

10.3

81.8

1.09

5.41

79.8

13.7

cKit

Sca-1

**A**

8

6

4

2

0

10

CD34

CD16/32

**GMP**

2.33

**CMP**

96.5

**MEP**

0.92

**GMP**

51.6

**CMP**

44.2

**MEP**

2.84

% Lin-cKit+Sca-1-

CMP

GMP

MEP

100

60

40

20

0

80

120

DNM^f/-^Vav^+/-^

Control

*

*

**B**


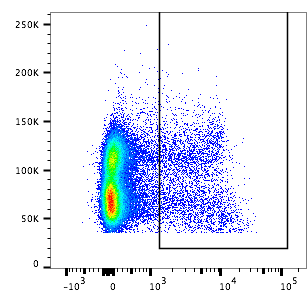

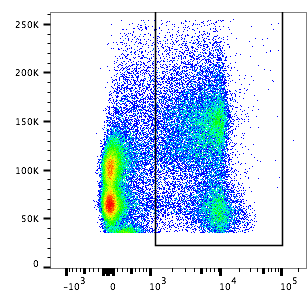


80

60

40

20

0

CD11b+ Cells

FSC

CD11b

34.3

5.26

100

*

**C**


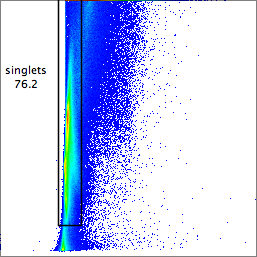

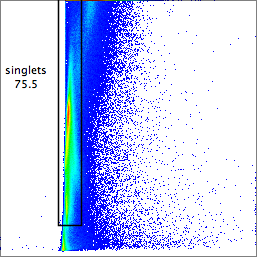

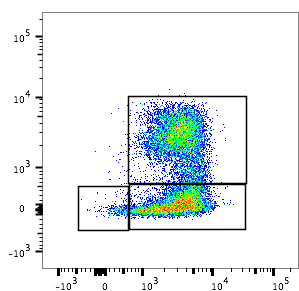


myeloid sub-progenitors

CD16/32

CD34


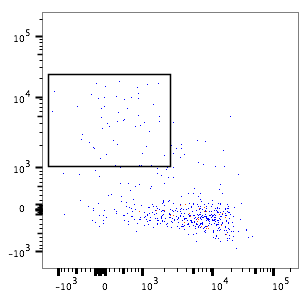


LT-HSCs

CD48

CD150


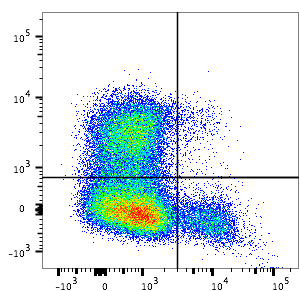


LSK

Sca-1

cKit

**D**

**E**

total myeloid

progenitors


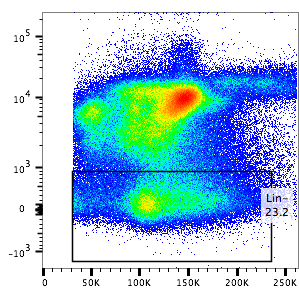

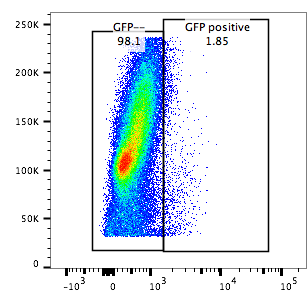

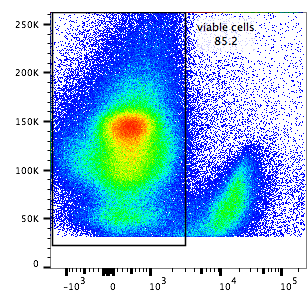


Lin-

GFP-

FSC

Lin BV711

Viable cells

Count


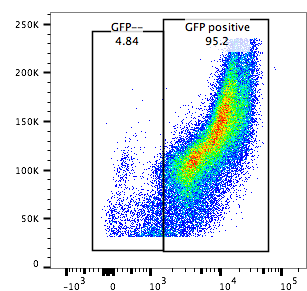

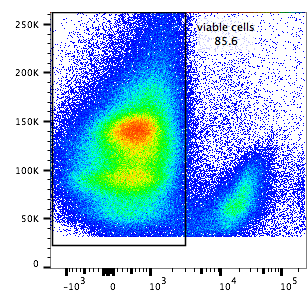


FVS620

FSC

Count

FSC

Lin BV711

GFP+


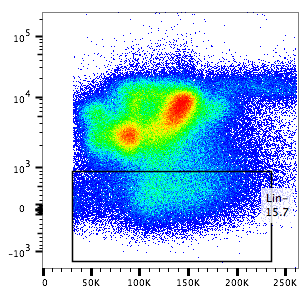


**Controls**

**DNM^f/-^Vav^+/-^**

GFP


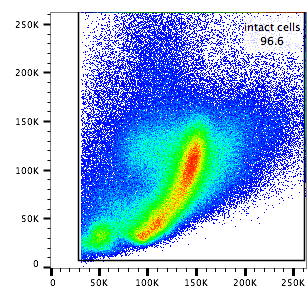


SSC

Intact cells

FSC

SSC


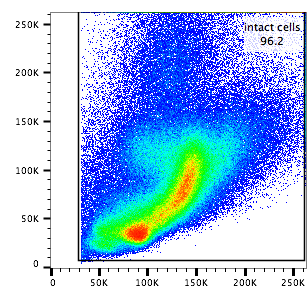


Doublet discrimination

SSC-W

FSC-A

FSC-A

**Supplementary Figure 2: Frequency of HSPCs and myeloid cells in the spleen of DNM^f/-^Vav^+/-^ and control mice at 15-18 months.** HSPC analysis of spleen cells harvested from DNM^f/-^Vav^+/-^ and control mice euthanized at 15-18 months. Representative dot plots showing DNM^f/-^Vav^+/-^ LSK cells **(A),** total myeloid progenitors **(A),** myeloid sub-progenitors **(B),** and mature myeloid cells **(C)** compared to controls are shown in left panels. Quantification of data from five mice (n=5) is shown in right panels. Data is representative of 2 independent experiments. Statistical significance was determined by a two-tailed, unpaired *t-*test (* *P≤* 0.05). **D.** Gating Strategy used to identify DNM^f/-^Vav^+/-^ HSPCs. Bone marrow cells and splenocytes were harvested from DNM^f/-^Vav^+/-^ and control mice euthanized at various time points. Cells were stained with a fixable viability stain and HSPC markers, then analyzed by flow cytometry. **E.** DNM^f/-^Vav^+/-^ LSK cells were identified by gating on the GFP+ fraction of Lin-negative cells, followed by Sca-1+cKit+ cells. Total myeloid progenitors were identified as Sca-1-cKit+ cells. Myeloid progenitor populations were identified by gating on the GFP+ fraction of Lin-negative cells, followed by cKIt+Sca-1- cells and finally CD16/32 vs CD34: CMP (Lin-Sca-1-cKit+CD16/32-CD34+), GMP (Lin-Sca-1-cKit+CD16/32+CD34+) and MEP (Lin-Sca-1-cKit+CD16/32-CD34-). LT-HSCs were identified by gating on the GFP+ fraction of Lin-negative cells, followed by Sca-1+cKit+ cells and finally CD150+CD48- cells. HSPCs from control mice were identified by gating on the GFP- fraction of Lin-negative cells, followed by the same gating strategies used to identify DNM^f/-^Vav^+/-^ HSPCs.


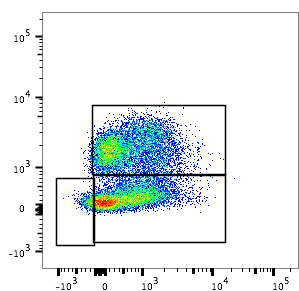

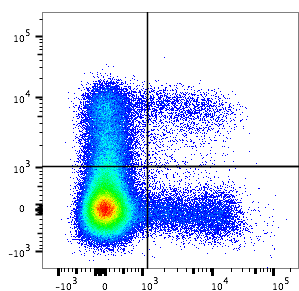

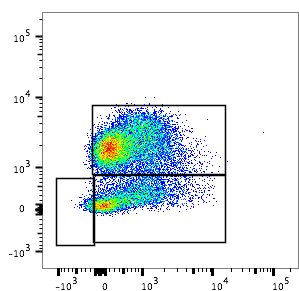

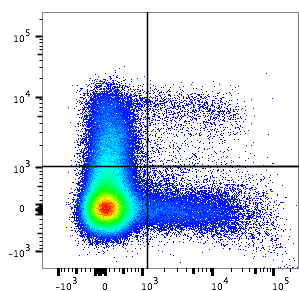


% Lin-

Myeloid

progenitors

LSK

**Control**

**DNM^f/-^Vav^+/-^**

0.76

6.03

11.2

82.0

1.33

13.5

80.3

4.80

cKit

Sca-1

0

10

20

30

40

100

CD34

CD16/32

**GMP**

46.0

**CMP**

49.7

**MEP**

4.08

**GMP**

67.8

**CMP**

29.5

**MEP**

1.97

% Lin-cKit+Sca-1-

CMP

GMP

MEP

0

20

60

80

40

**A**

**B**

*

DNM^f/-^Vav^+/-^

Control

**Supplementary Figure 3: Frequency of HSPCs in the BM of DNM^f/-^Vav^+/-^ and control mice at 6 months.** HSPC analysis of bone marrow cells harvested from DNM^f/-^Vav^+/-^ and control mice euthanized at 6 months. Representative dot plots showing DNM^f/-^Vav^+/-^ LSK cells **(A),** total myeloid progenitors **(A)** and myeloid sub-progenitor cells **(B)** are shown in comparison to control marrow cells. Quantification of data from three mice (n=3) is shown in right panels. Data is representative of 2 independent experiments. Statistical significance was determined by a two-tailed, unpaired *t-*test (* *P≤* 0.05)

# of colonies

0

5

10

15

40

120

3

2

1

Control Bone Marrow 1

DNM+/-Vav+/- Bone Marrow 1

Control Bone Marrow 2

Control Bone Marrow 3

DNM+/-Vav+/- Bone Marrow 2

DNM+/-Vav+/- Bone Marrow 3

80

**A**

Weeks

**Supplementary Figure 4: Myeloid colonies generated from DNM^f/-^Vav^+/-^ BM cells at 6 months.** **A**. Graph showing the number of myeloid colonies generated from the whole bone marrow of 6 month old DNM^f/-^Vav^+/-^ (n=3) mice compared to age matched control mice (n=3) for 3 weeks using a myeloid colony forming assay.

**Supplementary Figure 5: Analyses of peripheral blood and spleen cells from DNM^f/-^Vav^+/-^ mice aged to 18 months A**. At 12 months, GFP expression in the peripheral blood of DNM^f/-^Vav^+/-^ mice was evaluated using flow cytometry (n=18). **B.** In addition, the percentages of GFP-positive cells within the myeloid (CD11b+), B cell (B220+) and T cell (CD3+) compartments were also evaluated by flow cytometry, demonstrating variable GFP expression in the T cell compartment. **C.** DNM^f/-^Vav^+/-^ mice aged to 15-18 months were euthanized, spleens were harvested and cells were stained with B220, CD3 and MZ B-cell markers: sIgM and CD21. MZ B-cells were identified by gating on B220+CD3- cells (top) followed by sIgM+CD21+ cells (bottom) as shown in the representative dotplots. **D.** Quantification of data from five mice (n=5) is shown. Data is representative of 2 independent experiments. Statistical significance was determined by a two-tailed, unpaired *t-*test (* *P≤* 0.05).

# of colonies

Weeks

0

20

40

80

400

500

600

60

1

2

3

300

200

**B**

Control untransduced

DNM+/-Vav+/- untransduced

Control MIC

Control FLT3-ITD

DNM+/-Vav+/- MIC

DNM+/-Vav+/- FLT3-ITD

80

100


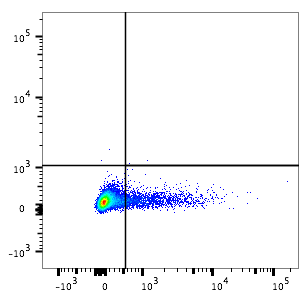

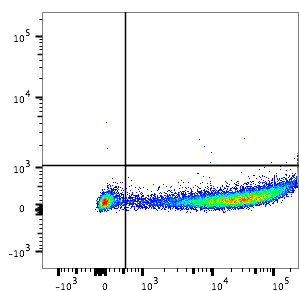

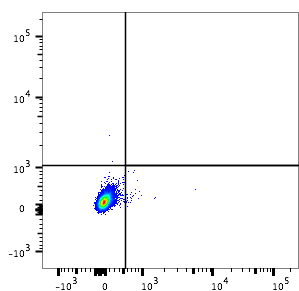

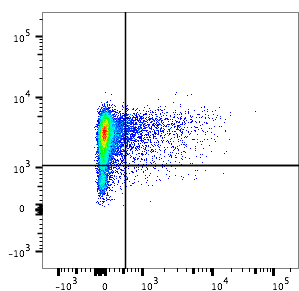

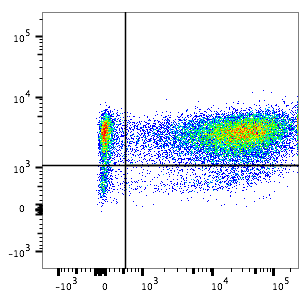

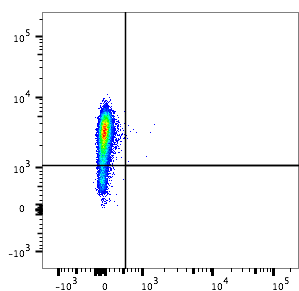


0.17

9.81E-3

17.0

82.3

GFP

77.8

14.2

2.81

5.20

mcherry

0

0.010

99.8

0.17

0.090

89.5

10.4

0

GFP

mcherry

Control FLT3 ^ITD^

DNM^f/-^Vav^+/-^ FLT3 ^ITD^

**A**

0.010

0.010

91.1

8.90

GFP

7.37

83.6

8.52

0.49

mcherry

**Untransduced**

**FLT3-ITD MIC**

**MIC**

**Supplementary Figure 6: Analyses of bone marrow cells from DNM^f/-^Vav^+/-^FLT3^ITD^ mice. A**. Whole bone marrow cells were harvested from DNM^f/-^Vav^+/-^ or control mice at euthanasia, Lineage negative cells were obtained and transduced with FLT3^ITD^ MSCV-Ires-mCherry (MIC) construct then transplanted into CD45.1 recipients. Representative flowplots showing initial transduction efficiency of cells on day of transplant: comparison of untransduced cells, cells transduced with empty vector or transduced with FLT3^ITD^ **B**. Graph showing the number of colonies generated from DNM^f/-^Vav^+/-^FLT3^ITD^  Lin-negative cells (grey) compared to control FLT3^ITD^ cells (green) over time. Controls included in the experiment are: control cells that are untransduced (black); control cells transduced with MIC only (red); DNM^f/-^Vav^+/-^ cells that are untransduced (blue); DNM^f/-^Vav^+/-^ cells transduced with MIC only (white).

**Supplementary References:**

1. Tu L, Fang TC, Artis D, Shestova O, Pross SE, Maillard I, et al. Notch signaling is an important regulator of type 2 immunity. The Journal of experimental medicine. 2005;202(8):1037-42.

2. Georgiades P, Ogilvy S, Duval H, Licence DR, Charnock-Jones DS, Smith SK, et al. VavCre transgenic mice: a tool for mutagenesis in hematopoietic and endothelial lineages. Genesis. 2002;34(4):251-6.

3. Grieselhuber NR, Klco JM, Verdoni AM, Lamprecht T, Sarkaria SM, Wartman LD, et al. Notch signaling in acute promyelocytic leukemia. Leukemia. 2013;27(7):1548-57.
